# The feasibility of measuring the activation of the trunk muscles in healthy older adults during trunk stability exercises

**DOI:** 10.1186/1471-2318-8-33

**Published:** 2008-12-04

**Authors:** Edwin Y Hanada, Cheryl L Hubley-Kozey, Melissa D McKeon, Sarah A Gordon

**Affiliations:** 1 Department of Medicine, Capital District Health Authority and Dalhousie University, Halifax, Canada; 2 School of Physiotherapy, Dalhousie University, Halifax, Canada; 3 School of Biomedical Engineering, Dalhousie University, Halifax, Canada

## Abstract

**Background:**

As the older adult population increases, the potential functional and clinical burden of trunk muscle dysfunction may be significant. An evaluation of risk factors including the impact of the trunk muscles in terms of their temporal firing patterns, amplitudes of activation, and contribution to spinal stability is required. Therefore, the specific purpose of this study was to assess the feasibility of measuring the activation of trunk muscles in healthy older adults during specific leg exercises with trunk stabilization.

**Methods:**

12 asymptomatic adults 65 to 75 years of age were included in the study. Participants performed a series of trunk stability exercises, while bilateral activation of abdominal and back extensor muscles was recorded by 24 pairs of Meditrace™ surface electrodes. Maximal voluntary isometric contractions (MVIC) were performed for electromyographic (EMG) normalization purposes. EMG waveforms were generated and amplitude measures as a percentage of MVIC were calculated along with ensemble average profiles. 3D kinematics data were also recorded, using an electromagnetic sensor placed at the left lateral iliac crest. Furthermore, a qualitative assessment was conducted to establish the participant's ability to complete all experimental tasks.

**Results:**

Excellent quality abdominal muscle activation data were recorded during the tasks. Participants performed the trunk stability exercises with an unsteady, intermittent motion, but were able to keep pelvic motion to less than 10°. The EMG amplitudes showed that during these exercises, on average, the older adults recruited their abdominal muscles from 15–34% of MVIC and back extensors to less than 10% of MVIC. There were similarities among the abdominal muscle profiles. No participants reported pain during the testing session, although 3 (25%) of the participants reported delayed onset muscle soreness during follow up that was not functionally limiting.

**Conclusion:**

Older adults were able to successfully complete the trunk stability protocol that was developed for younger adults with some minor modifications. The collected EMG amplitudes were higher than those reported in the literature for young healthy adults. The temporal waveforms for the abdominal muscles showed a degree of synchrony among muscles, except for the early activation from the internal oblique prior to lifting the leg off the table.

## Background

Biomechanical research has demonstrated the role of trunk muscle activation during functional activities and exercise [1]. The significance of the trunk muscles for spinal stability has been previously established in working-age adults, and has been linked to prevention and rehabilitation of low back pain (LBP) [2]. In particular, endurance and coordination of trunk muscle activity are key characteristics to maintain the stability of the spine, and therefore decrease the effects of low back pain [1]. Furthermore, it has been demonstrated that trunk muscle activity occurs during locomotion, and while the precise role is not well understood, the evidence suggests that the trunk muscles fulfill a critical role in the dynamic control of posture [3]. A link between trunk muscle function, low back pain, and physical function in older adults has been reported [4]. It is clear that both the abdominal and the back extensor muscles are key components that contribute to spinal stability[1] and functional stability [3]. Given these interrelationships and the desire to prevent falls in this group, there is a need for better assessments of trunk muscle activation in older adults.

LBP is one of the most common medical disorders in older persons, affecting up to half of those over 65 years of age [5]. In the Iowa 65+ Rural health study, LBP was reported by 23.6% of older women, and by 18.4% of older men in the year prior to the survey [6]. There is an enormous health care cost for LBP in older adults, as nearly 75% of persons with this disorder may utilize medical and chiropractic services, while 25% have at least one hospitalization directly related to LBP, and 5% of participants have low back surgery [6]. Furthermore, 15 to 40% of the elderly respondents reported some type of disability, in the form of limitation in walking, sitting, bending over, and performing household chores, while 21% of the participants attributed sleep disturbance to the LBP [6].

In older adults, trunk extensor strength declines more quickly with aging than does appendicular strength [7]. This loss of strength in axial muscles has been associated with kyphosis [8]. Research has revealed that older adults have a delayed response in the onset of trunk muscle activation, a reduction in amplitude in paraspinal muscle firing, and smaller stretch reflexes in response to dynamic stability perturbations [9]. Furthermore, in a cross-sectional analysis of older adults, through multivariate models, isometric trunk extensor strength was demonstrated to be predictive of maximal walking speed among women, but not men [10]. A more recent study demonstrated that higher fat infiltration around the spine in persons aged 70 to 79 years was associated with reduced functional capacity as reflected by compromised balance [4]. Finally, Hwang et al. (2008) has confirmed that feed-forward responses of the paraspinal muscles are compromised in older adults based on response times reported for expected and unexpected perturbations.

With the older adult population (65+ years) being a fast growing age group in North America, we should expect to see the incidence of LBP and functional decline increase. An evidence-based approach to treatment is required to help mitigate the potential for spiraling health care costs and increased disability. The potential functional and clinical burdens of trunk muscle dysfunction in this population will be significant. Therefore, further evaluation of risk factors is required, including the impact of the trunk muscles with respect to their temporal firing patterns, amplitude of activation, and their contribution to spinal stability. Methods for measuring trunk muscle function have previously been established for younger adults [11,12], but very little is known about these methods in the older adult population. This study will focus on a leg-loading exercise that requires the abdominal muscles to respond to minimize lumbo-pelvic motion [11,13,14].

The long term goal is to improve diagnostic assessment tools for the "trunk" stabilizing muscles, and to better understand how impairments such as LBP affect the neuromuscular responses, so that we may improve our understanding of therapeutic exercises used in the management of LBP, especially in the older population. The specific objectives of this study were i) to assess the feasibility for measuring the activation of the trunk muscles in an older adult population, ii) to determine the safety of a method for measuring co-activation of trunk muscles, and iii) to quantify trunk muscle activation patterns during a leg-loading task that has been used as both an assessment tool and a therapeutic exercise.

The original protocol was used to investigate neuromuscular patterns of working-age adults (20–50 yrs) during a dynamic exercise stability test [11] that was designed to challenge spinal stability. Previous work has illustrated that those with LBP respond to the demands of dynamic challenges differently than those without LBP [13,15-18]. Although there may be serious consequences of neuromuscular impairments of the trunk muscles, including its effects on LBP and mobility in older adults, the authors are not aware of any published study that focuses on the characterization of trunk muscle activation of older adults during a therapeutic exercise.

## Methods

### Participants

Sixteen asymptomatic healthy adults between 65 to 80 years of age were recruited through word of mouth to participate in the study. Participants were excluded if they had a history of LBP, previous abdominal or back surgery, previous spinal fracture, or any other major musculoskeletal, cardio-respiratory, or neurological condition. Throughout the screening process, 4 individuals (2 males, 2 females) were excluded due to medical problems (arrhythmia, coronary artery disease, osteoporosis), leaving 12 individuals (7 males, 5 females) to participate in the study.

### Screening & Questionnaires

Prior to participation, all individuals were required to read and sign an informed consent approved by the Capital Health District's Research Ethics Board. All participants were interviewed to determine any medical conditions that would prevent their participation in the study, their health status, and information regarding participation in regular abdominal exercise and fitness routines.

Participants were asked to attend two testing sessions: the first one being 30 minutes in duration, and the second session 2 hours. During the first session, a postural and neurological assessment was conducted by a physiotherapist to screen for any obvious fixed abnormal spinal postures (kyphosis, lordosis, or scoliosis) and lower extremity neuromuscular deficits (myotomal strength, reflexes, and dermatomal sensation). Furthermore, a mental status examination was performed to ensure adequate cognitive ability to participate in the research study (score > 23) [19]. Standard demographic data was collected from each individual, including age, sex, occupation, and anthropometric data such as mass (in kilograms), height (in centimeters), and waist circumference (in centimeters). Body mass index (BMI) was calculated from the height and mass measures. Demographic data is found in Table 1.

**Table 1:** Subject demographic data displayed as means and standard deviations for age, mass, height, BMI, and waist girth.

	**Age** **(yrs)**	**Mass** **(Kg)**	**Height** **(cm)**	**BMI** **(Kg/m^2^)**	**Waist girth** **(cm)**
**Group**	68.7 *(± 3.5)*	79.1 *(± 14.8)*	170.0 *(± 9.3)*	27.2 *(± 3.5)*	91.2 *(± 13.2)*

### Trunk Stability Exercise Protocol

Three levels of a trunk stability exercise protocol [11,16] were used to progressively challenge the individual's stabilizing system without placing high mechanical loads on the lumbar vertebrae. During the first testing session, participants were shown and given a verbal description of each exercise and told to practice each of the 3 levels 5 times on 3 separate occasions before returning for their second testing session.

During the second testing session the trunk stability exercises were performed in random order to minimize effects of fatigue and learning. Each exercise was repeated three times with a one-minute rest between trials. The start and end position for each exercise level was standardized, with participants lying in a supine position and knees flexed to 90°. Participants were given instructions to activate the abdominal muscles prior to lifting or extending their legs.

**Figure 1:**
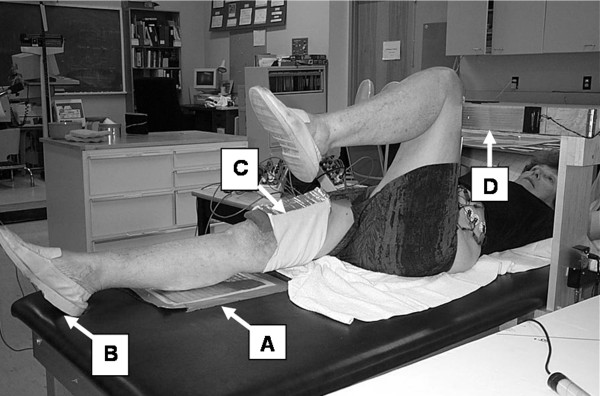
**Level 2 of the exercise progression as per the description in the text**. The heel slides along the table until the right knee is fully extended. Electronic switches are located on the right foot [B] which contacts with the metal plate located on the table [A] and the right thigh [C] which contacts with the wooden frame [D] to identify temporal events so that the motion may be divided into distinct phases. The total exercise was from foot off the bed to foot back on the bed. For levels 2 and 3, the knee extension phase was from knee off the wooden frame to knee back on the wooden frame.

In Level I of the exercise, participants were instructed to lift their right foot off of the table and flex the knee and the hip to 90° and have the thighs contact the wooden frame, then lift the left foot off to the same position, then lower the legs (left then right) to return to the starting position. Level 2 of the exercise protocol added a right knee extension phase after the left thigh came in contact with the wooden frame, as shown in Figure 1. The right heel slid along the table until the knee was fully extended, upon which the hip and knee were then flexed and returned to the 90° hip angle position, and subsequently both legs were lowered to the starting position (left then right). Level 3 repeated the sequence of level 2, except that the foot did not touch the bed until the knee was fully extended [11]. At full extension, only the heel was briefly tapped on the bed before the right leg was returned to the wooden frame without further contact with the bed.

Electronic switches were placed on the right thigh and the plantar surface of the right foot to identify temporal events so that the motion could be subdivided into distinct phases of lift, extend, and lower. The total exercise was defined from right foot off the bed to right foot back on the bed. For levels 2 and 3 the extension phase was from knee off the wooden frame to knee back on the wooden frame

### Normalization Exercises

Normalization exercises followed the trunk stability exercises to elicit the maximal voluntary isometric contraction (MVIC). Participants performed a randomized series of isometric exercises in an attempt to recruit all motor units for amplitude normalization purposes. Exercises for each muscle site were chosen based on previous work [20-22] and muscle functional testing procedures [23]. While other methods of normalization have been used to answer specific questions [24], normalizing to MVIC is an acceptable standard for comparison but the results need to be interpreted within the limitations of this procedure [25]. The idea of using a variety of exercises is important to elicit maximal contractions from the abdominal muscles [21,26] and providing feedback and motivation to the participant are necessary to increase the potential for eliciting a maximum effort [27]. The exercises included a resisted sit-up, back extension, lateral bend and trunk rotation and all have been used in previous studies on asymptomatic controls and those with low back pain [11,13,16]. In all exercises, the participants were stabilized using Velcro straps and applied manual resistance to minimize potential movement. Each exercise was repeated twice with a two-minute rest between exercises to minimize fatigue.

### Electromyography (EMG)

Surface EMG (3-AMT-8, Bortec™, Canada) was recorded from 24 muscle sites on the abdomen (12) and back (12) while participants performed the trunk stability exercise tasks. The electrode placements were selected to provide information on bilateral sites for a comprehensive set of abdominal and back extensor muscle sites [28] and the sites are consistent with de Seze's recent work [29]. Meditrace™ Ag/Ag Cl surface electrodes (10 mm diameter, bipolar configuration 30 mm centre-to-centre) were attached after standard skin preparation to the left and right sides of the: i) lower rectus abdominis (LRA): centered on the muscle belly midway between the umbilicus & the pubis [30]; ii) upper rectus abdominis (URA): centered on the muscle belly midway between the sternum & the umbilicus [30]; iii) external oblique anterior fibres (EO1): over the 8^th ^rib adjacent to the costal cartilage [31]; iv) external oblique lateral fibres (EO2): 15 cm lateral to the umbilicus oriented at 45° [21]; v) external oblique posterior fibres (EO3): midpoint between the lowest part of the ribcage and the iliac crest and vi) internal oblique (IO): centered in the triangle formed by the inguinal ligament and lateral border of the rectus abdominis sheath and the line between the anterior superior iliac spine [32]. For the back extensors, electrodes were attached at. four sites on the erector spinae: vii and viii) lumbar longissimus 3 cm from the spinous process at lumbar level 1 (L1–3) and level 3 (L3-3) [33-35]; ix) and x) lumbar iliocostalis 6 cm from the spinous process at lumbar level 1 (L1–6) and level 3 (L3–6) [33-35]; xi) multifidus 2 cm from spinous process at lumbar level 5 (L5-2) [22]; and xii) a site over the quadratus lumborum 8 cm from the spinous process at lumbar level 4 (L4–8) [36,28] that was verified in pilot work on cadavers for a total of 24 recording sites. These placements were used as a general guide and minor adjustments were made for different body sizes.

There were some difficulties in locating landmarks for EMG electrode placement and electrode adherence due to skin movement. The electrodes were placed during standing and then verified using palpation and resisted exercises to ensure proper placement and to assess cross talk while participants were in the supine position. The exercises for verification attempted to isolate the specific muscle site and were based on manual muscle testing [23,37]. The back extensor sites were stable between the standing and lying position. However, minor adjustments were made for a small number of subjects in which movement due to adipose tissue occurred between standing and lying supine for some abdominal sites. The normalization exercises were used as a validation for proper placement, since exercises were aimed at recruiting specific abdominal muscles and minimizing activity in other muscles.

### Motion Capture

**Figure 2:**
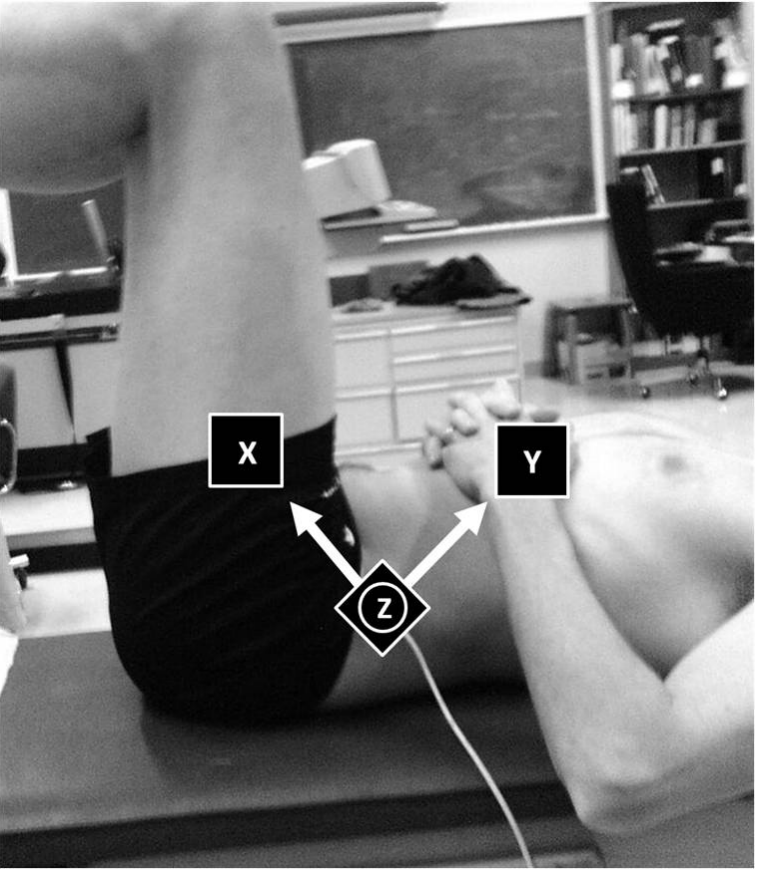
**Motion capture**. The Flock of Birds Motion Capture™ sensor was placed on the antero-superior portion of the left lateral iliac crest. Note that the z-axis is positioned perpendicular to the sensor. This sensor records motion with respect to a fixed global reference and not an anatomical reference.

A Flock of Birds Motion Capture™ system was used to record the angular motion of the pelvis during the tasks. A sensor was placed on the antero-superior portion of the left lateral iliac crest. The sensors detected changes in 3 D motions with respect to a global reference providing an overall measure of motion, but the measurements can not be related directly to an anatomical reference (Figure 2). The motion data was synchronized to the EMG data via the external sensors with each motion profile normalized to 100% time. The motion data was used to ensure minimal movement of the trunk and pelvis and to confirm that participants were able to maintain their lumbar pelvic position of a neutral spine throughout all tasks.

### Data processing and analysis

Root mean square amplitudes were calculated for the total exercise for all three levels and for the extension phase for levels 2 and 3. These amplitudes were normalized to the % MVIC [11,38]. In addition, the raw EMG signals were full-wave rectified and low pass filtered at 6 Hz using a second order recursive Butterworth filter to yield a linear envelope waveform. The waveforms were time normalized for the total exercise and then amplitude normalized to the %MVIC. Ensemble average waveforms were calculated for each muscle and a coefficient of variation for the waveforms [39].

The maximum difference in angular motion around the three axes was calculated during the entire exercise for all three levels and for the leg extension phase of levels 2 and 3.

## Results

### Participants

Participants were recruited from the local community and had several years of university/college education (oceanographer (2), teacher/professor (3), social worker (2), supervisor, superintendent, engineer, librarian, and physician). Four older adults were excluded after the first session due to health problems pertaining to the health screening questionnaire, as previously detailed in the methods section. All 12 participants (7 men and 5 women) participated in both testing sessions, leaving a 0% drop-out rate.

### Trunk Stability Exercise

Throughout the first session, the older adults were found to have difficulty following and remembering instructions. This problem was resolved by simply demonstrating the actions, in addition to verbalizing the instructions. It was found that the older adults performed the exercises with intermittent, jerky motions as opposed to a smooth motion. An attempt was made to correct this throughout the first session and participants were instructed to practice these smoothed movements at home. Subsequently, performance was improved from a qualitative perspective during the second session.

### Normalization and Validation Exercises

Three participants (25%) were instructed to perform the normalization exercises at a sub-maximal effort given their history of hypertension. Furthermore, it was necessary to remind the participants to breathe throughout the trunk muscle contractions, due to the possibly harmful increase in intra-thoracic/intra-abdominal pressure associated with the Valsalva maneuver. The male participants reported "not being able to push as hard if they did not hold their breath". In contrast, it is uncertain whether the women exerted a true maximum effort based on self-report and the observed absence of visible exertion. During normalization trials the men were not hesitant to express fatigue and the need to rest between trials, but the women often reported "feeling fine" and being "ready to go again" immediately following each trial. However, in order to minimize the effects of fatigue, the standard rest time (120 sec) was applied for both groups.

### Motion

Participants were able to minimize pelvic motion during the total exercise to an average less than 10° and to less than 5° during the extension phase of the trunk stability tasks for all three axes as shown in Table 2.

**Table 2:** Maximum motion (in degrees) about the sensor for the total exercise for all 3 exercise levels and for the leg-extension phase of levels 2 and 3.

	**Total**			**Leg-extension**		
	**Yaw**	**Pitch**	**Roll**	**Yaw**	**Pitch**	**Roll**
**Level 1**	5.7 *(± 3.4)*	4.7 *(± 2.2)*	6.8 *(± 3.7)*	--	--	--
**Level 2**	7.0 *(± 3.5)*	7.5 *(± 3.8)*	9.5 *(± 5.1)*	3.1 *(± 1.9)*	4.0 *(± 2.7)*	4.3 *(± 3.3)*
**Level 3**	6.8 *(± 2.9)*	7.3 *(± 3.6)*	8.9 *(± 4.6)*	2.8 *(± 1.2)*	3.6 *(± 2.5)*	3.3 *(± 2.6)*

Means *(SD) *are displayed for *Yaw *(motion about the Z-axis), *Pitch *(motion about the y-axis), *Roll *(motion about the x-axis) with respect to a global reference.

### Electromyography

**Figure 3:**
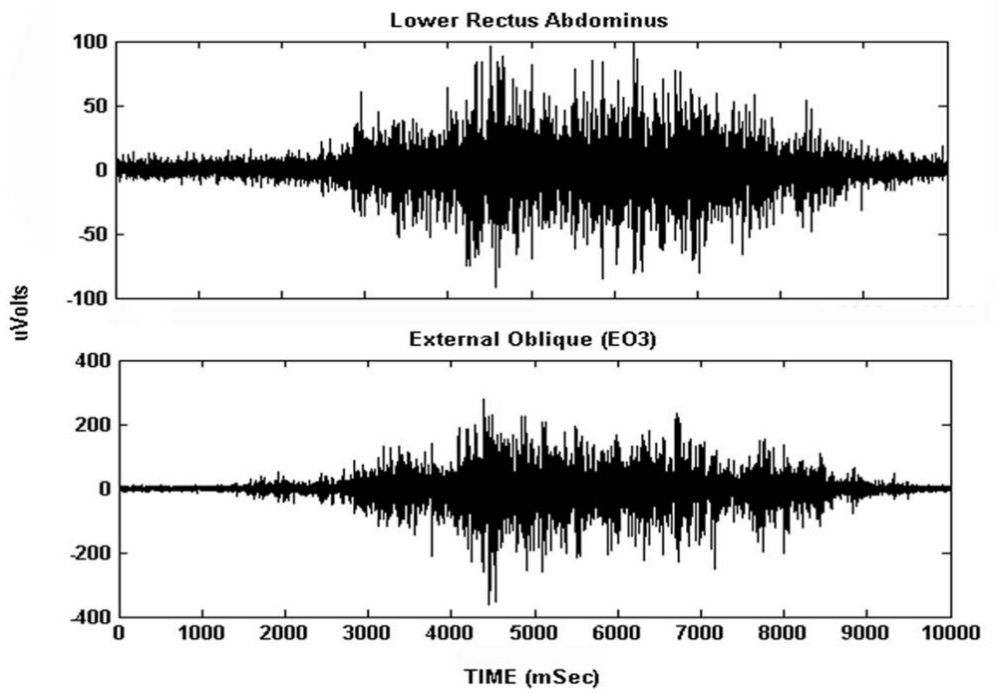
**Raw EMG data**. Examples of the raw EMG signals from the lower rectus abdominus [LRA], and external oblique [EO3] during level 3 of the exercise progression. This provides an indication of the quality of the data.

**Figure 4:**
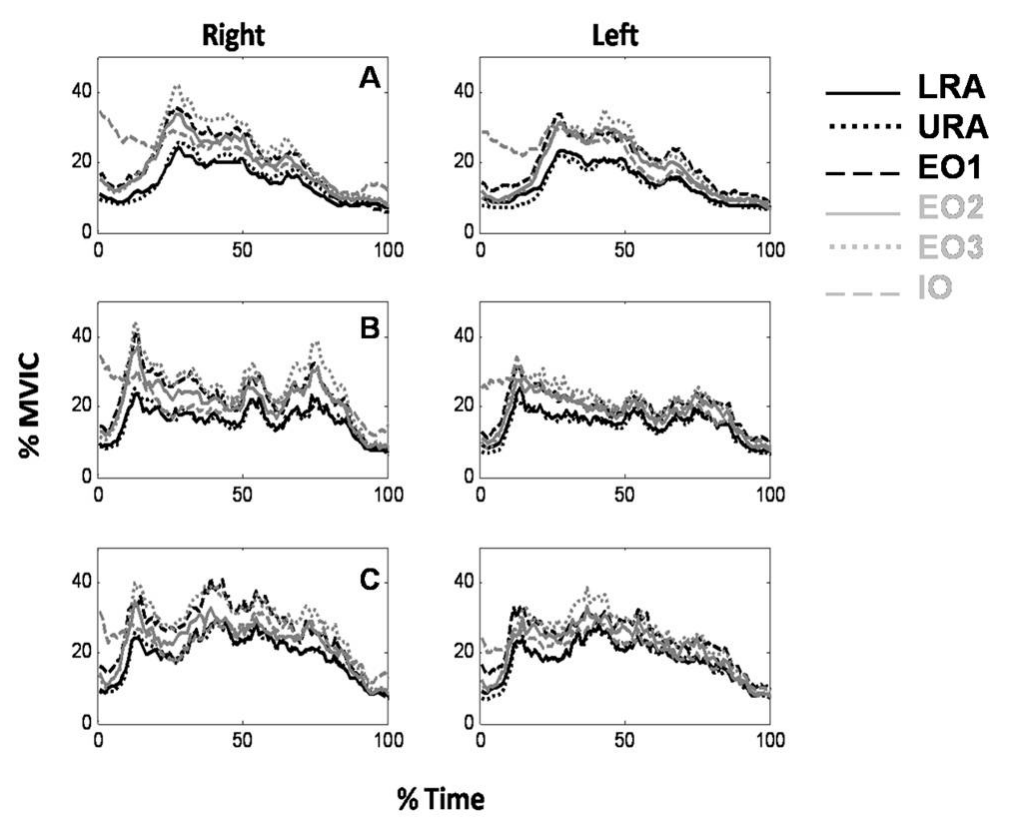
**Sample ensemble average waveforms**. Sample ensemble average waveforms normalized to %MVIC for the 6 abdominal muscles during 3 levels of the exercise progression. [A] Level 1 [B] Level 2 [C] Level 3. Note that three participants did not produce maximal activations during normalization exercises. Coefficients of variation are in Table 4.

Although there were some challenges to the fixation of the electrodes to the participants' skin that included: 1) difficulty in locating landmarks for EMG electrode placement because of loose skin and excess adipose tissue, and 2) electrode adherence to the skin during movement, all EMG signals were of high quality, with excellent signal to noise ratios. Examples of the abdominal signals are illustrated in Figure 3. There was low amplitude noise from the magnetic motion sensors, but this had no affect on the abdominal muscles and had no visible effect on the back extensor muscles. Pilot work showed that the low level noise decreased the further the sensor was from the transmitter, and that it was negligible when at a distance greater than 60 cm. For the exercise tasks, this distance was greater than 60 cm. The mean amplitude for all 24 muscle sites is presented in Table 3. As expected, participants activated back extensor muscles to low amplitudes, with all sites less than 10% MVIC. The abdominal muscles were recruited to amplitudes from 15% to 35% MVIC (lower amplitudes for level 1, higher amplitudes for level 3). The ensemble average profiles are in Figure 4 for levels 1–3. The coefficients of variation for these waveforms are found in Table 4. These profiles illustrate how the muscles respond to the changes in loading throughout the trunk stability tasks. Of note is the higher bilateral internal oblique amplitude initially for all exercise levels.

**Table 3:** Mean *(SD) *RMS amplitudes normalized to a maximal effort contraction (%MVIC) for the total exercise for level 1 and for the leg extension phase of levels 2 and 3.

		**Abdominal Muscles**			**Back Extensor Muscles**	
		**Right**	**Left**		**Right**	**Left**
**Level 1** **Total Exercise**	**LRA**	15.6 *(± 6.6)*	15.6 *(± 5.4)*	**L1–3**	6.1 *(± 2.2)*	5.7 *(± 1.8)*
	**URA**	16.4 *(± 10.3)*	14.5 *(± 6.5)*	**L1–6**	8.1 *(± 2.5)*	8.1 *(± 2.7)*
	**EO1**	22.5 *(± 13.4)*	21.6 *(± 6.8)*	**L3-3**	5.2 *(± 1.2)*	4.6 *(± 1.4)*
	**EO2**	20.7 *(± 10.5)*	20.0 *(± 12.9)*	**L3–6**	5.4 *(± 2.1)*	5.3 *(± 1.7)*
	**EO3**	24.9 *(± 9.1)*	21.5 *(± 9.2)*	**L4–8**	8.2 *(± 2.3)*	7.5 *(± 3.7)*
	**IO**	22.4 *(± 14.1)*	22.4 *(± 9.4)*	**L5-2**	7.2 *(± 2.7)*	7.1 *(± 2.6)*

**Level 2** **Leg Extend**	**LRA**	18.4 *(± 7.4)*	17.2 *(± 5.2)*	**L1–3**	5.2 *(± 1.9)*	5.3 *(± 1.6)*
	**URA**	17.4 *(± 8. 6)*	16.5 *(± 7.2)*	**L1–6**	6.6 *(± 1.8)*	8.1 *(± 3.2)*
	**EO1**	25.0 *(± 10.5)*	21.0 *(± 9.1)*	**L3-3**	4.9 *(± 1.8)*	5.2 *(± 2.7)*
	**EO2**	22.7 *(± 10.6)*	19.8 *(± 10.9)*	**L3–6**	4.9 *(± 2.4)*	6. 6 *(± 3.2)*
	**EO3**	27.6 *(± 8.5)*	22.9 *(± 9.2)*	**L4–8**	7.0 *(± 2.3)*	8.8 *(± 4.1)*
	**IO**	23.4 *(± 13.7)*	20.6 *(± 6.6)*	**L5-2**	7.0 *(± 3.0)*	7.2 *(± 2.7)*

**Level 3** **Leg Extend**	**LRA**	25.4 *(± 12.5)*	23.2 *(± 9.5)*	**L1–3**	5.5 *(± 2.4)*	5.5 *(± 2.1)*
	**URA**	24.9 *(± 14.4)*	23.4 *(± 13.2)*	**L1–6**	7.0 *(± 2.0)*	8.6 *(± 3.9)*
	**EO1**	33.3 *(± 13.0)*	28.5 *(± 15.2)*	**L3-3**	5.1 *(± 2.0)*	5.4 *(± 3.1)*
	**EO2**	27.3 *(± 11.1)*	26.8 *(± 19.9)*	**L3–6**	5.0 *(± 2.1)*	7.0 *(± 3.6)*
	**EO3**	33.8 *(± 9.9)*	30.4 *(± 17.9)*	**L4–8**	7.5 *(± 2.9)*	9.5 *(± 4.5)*
	**IO**	27.3 *(± 13.8)*	23.9 *(± 9.2)*	**L5-2**	7.1 *(± 3.4)*	7.4 *(± 3.1)*

**Table 4:** Coefficients of variation for ensemble average waveforms for levels, levels 1– 3 of the trunk stability exercise task.

		**Abdominal Muscles**			**Back Extensor Muscles**	
		**Right**	**Left**		**Right**	**Left**
**Level 1**	**LRA**	0.50	0.41	**L1–3**	0.43	0.40
	**URA**	0.67	0.53	**L1–6**	0.39	0.45
	**EO1**	0.72	0.42	**L3-3**	0.33	0.37
	**EO2**	0.57	0.61	**L3–6**	0.42	0.37
	**EO3**	0.48	0.47	**L4–8**	0.33	0.52
	**IO**	0.68	0.49	**L5-2**	0.45	0.41

**Level 2**	**LRA**	0.49	0.40	**L1–3**	0.39	0.36
	**URA**	0.59	0.53	**L1–6**	0.33	0.44
	**EO1**	0.60	0.49	**L3-3**	0.38	0.48
	**EO2**	0.56	0.59	**L3–6**	0.48	0.47
	**EO3**	0.43	0.44	**L4–8**	0.35	0.51
	**IO**	0.68	0.46	**L5-2**	0.45	0.39

**Level 3**	**LRA**	0.56	0.48	**L1–3**	0.45	0.42
	**URA**	0.61	0.60	**L1–6**	0.38	0.50
	**EO1**	0.51	0.52	**L3-3**	0.38	0.52
	**EO2**	0.50	0.69	**L3–6**	0.44	0.47
	**EO3**	0.39	0.55	**L4–8**	0.39	0.52
	**IO**	0.59	0.44	**L5-2**	0.48	0.44

### Pain

No participants reported pain during the testing, and all participants were able to complete the full protocol with some minor adjustments to the normalization exercises as previously discussed. Follow-up phone calls were made to all participants following the testing sessions. Three men reported mild delayed-onset muscle soreness, although they were not limited in their daily function.

## Discussion

Generally, older participants were able to perform the exercises necessary for surface EMG measurement of trunk muscle activation patterns without significant difficulty, much like their younger cohorts demonstrated in previous studies [11,16]. Most importantly, they were able to perform the normalization procedures and exercise tasks safely, and without any major adverse health sequelae. For the former, however, three participants were instructed not to perform maximal level exercises because of their hypertension. All three were males and their percent MVIC values were not the highest for the test exercise. It was assumed that they produced an effort that was within normal variability for maximum efforts. Discussion of the implications of this alteration in procedure is discussed below. The results have important positive implications for future studies that focus on the activation of trunk muscles by demonstrating the feasibility of collecting comprehensive electromyographic data during this standardized task in healthy older adults.

Modifications were made to the protocol for older persons, both to increase the margin of safety and to enhance the probability of success in performing the tasks. For example, during the normalization procedure, participants were constantly reminded to breathe throughout the isometric contractions in order to reduce the potential effect of exercise-induced high blood pressure from a continued Valsalva maneuver, as found in a previous study [40]. Although there were minor challenges in participants learning to perform the trunk stability exercises, the addition of a live demonstration of these tasks to the participants helped to overcome any major obstacles to older adults being able to successfully perform these tasks.

Adipose tissue in the older adult participants made it challenging for the investigators to properly palpate musculoskeletal landmarks, and ensure that the electrodes were secure. Shifting of the electrodes occurred during the tasks, and some of the electrodes were required to be readjusted. However, this could happen in younger participants with increased waist circumference, and is not a challenge strictly for the older cohorts. Nevertheless, despite the challenges in the older adults, the validation exercises confirmed that the electrodes were properly placed and quality electromyography signals were recorded from the trunk muscles.

Excellent quality data was successfully recorded in the older adults during the leg-loading exercises. It was important that the older adults performed the exercise correctly and the motion data supports that they were able to minimize pelvic motion during performance of the exercise. The recorded motions gave an overall assessment of the pelvic motion with respect to a global reference and all angular displacement differences were less than 10 degrees. The older adults did not produce high levels of activity in the back extensor sites (< 10% MVIC) during performance of the exercise although they were higher than reports for younger adults [11,16]. The results also showed a degree of symmetry between the left and the right abdominal muscle sites, although a few (3/12) abdominal muscles had greater than a 4% MVIC difference between sides for the single-leg extension exercises i.e. EO1 for level 2 and 3 and EO3 for level 2. The increase in amplitude of activation from level 1–3 is consistent with reports for younger adults. However, the abdominal muscle amplitudes were slightly higher than amplitudes reported for younger adults [11,16]. This may reflect the decrease in strength for an older adult [41] and subsequently their need to work at a higher percentage of MVIC to perform the same task. As previously mentioned, three participants did not perform maximal exercises for normalization purposes due to their heart conditions. This could also explain the slightly higher amplitude for the older adults compared to the literature for young adults [11,16]. While it is recognized that there are limitations in using maximum voluntary activations for normalization purposes, and others have explored different normalization procedures [24,42], the results do provide an indication of the amplitude of muscle activity during the exercise compared to their voluntary maximum and allows for comparisons among muscles [43]. There will be variability in how forceful individuals voluntarily contract their muscles, and on average it has been shown to be 94% of the maximum amplitude produced during a burst superposition for the knee extensors [27]. In the present case, the average difference between including those that did sub-maximum and not including them was 0.76% MVIC. The results show that for all exercise levels, the activation did not exceed 35% of MVIC, so at best these exercises produced moderately low activation amplitudes in this group. It was also noted that in studies that use MVICs for normalization purposes, that comparison among studies show consistency in amplitudes and allows for conclusions to be drawn with respect to the demand that an exercise has on recruiting specific muscles [44]. Therefore the implication of sub maximal efforts is that the amplitudes as a percentage of maximum for the test exercises were overestimated and the exercises elicited even lower percent MVICs than presented in table 2.

Since the amplitudes recruited for the abdominal muscles were less than 35% of MVIC, this indicated that even the highest exercise level would not suffice as a strength training protocol. This conclusion is based on the American College of Sport Medicine guidelines for exercise training in older adults [41]. However, the exercises appear to be beneficial for recruiting trunk muscles, especially the abdominal muscles that are not normally recruited in every day activities. Furthermore, the coordination of trunk muscle activity that would be encouraged by performing the exercises in this protocol are considered important for spinal stability [44]. The coefficients of variation for this older adult are similar to those reported for younger asymptomatic subjects, but lower than those reported for young adults [16] with chronic low back pain [13].

The profiles illustrate the participants activated their abdominal muscles to a relatively consistent level throughout the exercise and did not have specific responses to the different phases within the exercise (i.e. a constant co-activation among the synergists rather than a response to the different loading associated with the leg lift/lower phases and leg extension phase). While one may argue that the instructions were specific to stabilize the pelvis resulting in co-activity, an asynchronous firing pattern was previously reported for those who had low back pain who were given the same instructions [15]. The higher level of activation of the internal oblique prior to the second leg lifting from the table suggests that it has an important role initially for stability, whereas the other muscles respond to the leg lifting portion of the exercise. This pattern for the internal oblique may have been a result of the instructions to stabilize the pelvis prior to lifting the foot off of the table.

There were several limitations regarding the generalizability of this study. Firstly, all of the participants were well-educated and therefore they may have been able to perform this task with a lesser degree of difficulty than an older adult who was not as well educated. This may be especially true, since some of the participants may have been more likely to engage in procedural, executive tasks in their daily work environment. Secondly, only 3 of the participants were over 70 years of age, and the extent that "older old" adults, such as those persons who were over 80 years of age, could perform the tasks was not determined in this study, and should be explored in future studies. Finally, these volunteers were healthy, and the degree to which older adults with back pain or mobility impairments could or would participate has not been determined. However, it is the authors' opinion that the protocol that was utilized could be sufficiently modified to accommodate for individuals with various impairments.

Another concern with the current protocol may be with the normalization procedure using maximal contractions. Kasman [43] acknowledged that while the participant with pathology may not be able to activate their muscles to a maximum amplitude, that it is still important to report the percentage of their maximal voluntary amplitude at which they are willing to recruit. Previous research demonstrated that even participants with joint pathology and pain are able to voluntarily activate their muscles to over 94% of their maximum when provided with learning and feedback [27]. While Lewek's study [27] was performed on lower limb muscles and not trunk muscles, it should be noted that these exercises have been used as a method for normalization for those with chronic low back pain with no adverse effects [13]. Given the lack of gold standard for EMG normalization, while it has its limitations and must be interpreted within these limitations, the MVIC is still the best standard for comparison among muscles [25,45].

Further research will be required to determine whether the protocol can be safely applied to an older adult population with various musculoskeletal disorders, such as LBP, again paralleling the research conducted on younger adults. A comparison study on younger sex-matched adults will help to elucidate the nature of the differences.

## Conclusion

Older adults were able to successfully complete the trunk stability exercise protocol to measure trunk muscle activation that was previously used for younger adults, with some minor modifications to the protocol with respect to instructions and normalization exercises. There were no adverse effects reported during or following the procedure. Quality EMG data from 12 bilateral trunk muscle sites were recorded during performance of the exercise allowing for study of co-activation among muscles during specific phases of the protocol. The older adults used low to moderate activation amplitudes to perform the trunk stability exercises. These results provide a foundation for future studies to evaluate the utility of EMG recordings of the trunk muscles during a limb-loading task as a clinical assessment tool for older adults with pathology. The paper established that this protocol for evaluating trunk muscle function was feasible, and that quality EMG records were obtained from this older adult group.

## Competing interests

The authors declare that they have no competing interests.

## Consent

A copy of the written consent is available for review by the Editor-in-Chief of this journal.

## Authors' contributions

EH: Designed study, manuscript preparation/editing and interpretation. CHK: Designed study, manuscript preparation/editing, analysis and interpretation. MM: Manuscript preparation, data analysis. SG: Recruited and collected data, manuscript editing.

## Pre-publication history

The pre-publication history for this paper can be accessed here:


